# Lirex: A Package for Identification of Long Inverted Repeats in Genomes

**DOI:** 10.1016/j.gpb.2017.01.005

**Published:** 2017-04-07

**Authors:** Yong Wang, Jiao-Mei Huang

**Affiliations:** Institute of Deep-Sea Science and Engineering, Chinese Academy of Sciences, Sanya 572000, China

**Keywords:** Lirex, Inverted repeats, Recombination, Stem-loop, Mismatch rate

## Abstract

Long inverted repeats (LIRs) are evolutionarily and functionally important structures in genomes because of their involvement in RNA interference, DNA **recombination**, and gene duplication. Identification of LIRs is highly complicated when mismatches and indels between the repeats are permitted. Long inverted repeat explorer (Lirex) was developed and introduced in this report. Written in Java, **Lirex** provides a user-friendly interface and allows users to specify LIR searching criteria, such as length of the region, as well as pattern and size of the repeats. Recombinogenic LIRs can be selected on the basis of **mismatch rate** and internal spacer size from identified LIRs. Lirex, as a cross-platform tool to identify LIRs in a genome, may assist in designing following experiments to explore the function of LIRs. Our tool can identify more LIRs than other LIR searching tools. Lirex is publicly available at http://124.16.219.129/Lirex.

## Introduction

Inverted repeats are reversely complementary sequences that are located adjacent to each other and mostly separated by an internal spacer in a genome. In single-stranded DNA or RNA, the inverted repeats may form a palindrome or a stem-loop structure. Mismatches between the repeats and the size of loop may affect stability of the stem-loop. Long inverted repeat (LIR) has >30 bp in length [1]. The significance of LIRs in genomes is reflected by their capacity of inducing DNA recombination [2], by their relatedness to gene deletion and amplification [3], [4], [5], and by their involvement in RNA interference [6], [7]. When a stem-loop structure is formed in mRNA by an LIR, the long and stable stem could be processed by Dicer to produce microRNAs (miRNAs) [8]. At present, there are approximately 2000 annotated miRNA genes (http://www.mirbase.org) [9]. The evolutionary significance of the LIRs in primates is still under hot debate. A previous study has identified primate-specific LIRs in long introns of the genes that are involved in development of nervous systems and detoxification in humans [10]. Some of them are suggested to be inducers of cancers [11], [12]. Thus, the functions of LIRs in humans are far more important than previously thought. It is likely that plenty of regulatory elements are hidden in intronic regions of primate genomes but have not been identified yet.

Several tools are available for searching LIRs. These include IRF (https://tandem.bu.edu/cgi-bin/irdb/irdb.exe), detectIR [13], EMBOSS palindrome tool (http://emboss.bioinformatics.nl/cgi-bin/emboss/palindrome), as well as integrated functions in BIOPHP (http://www.biophp.org) and MATLAB (https://www.mathworks.com/products/matlab.html). IRF and detectIR allow users to find LIRs with mismatches between inverted repeats. However, these methods can only detect limited number of LIRs due to simplicity of algorithms in dealing with mismatches. In this study, we introduce Lirex, a new tool, trying to detect more possible LIRs in genomes by searching the genomes exhaustively. We hope the release of Lirex will help to discover novel LIRs across a growing number of genomes and to revisit the significance of LIRs in evolution.

## Algorithm

**Figure 1:**
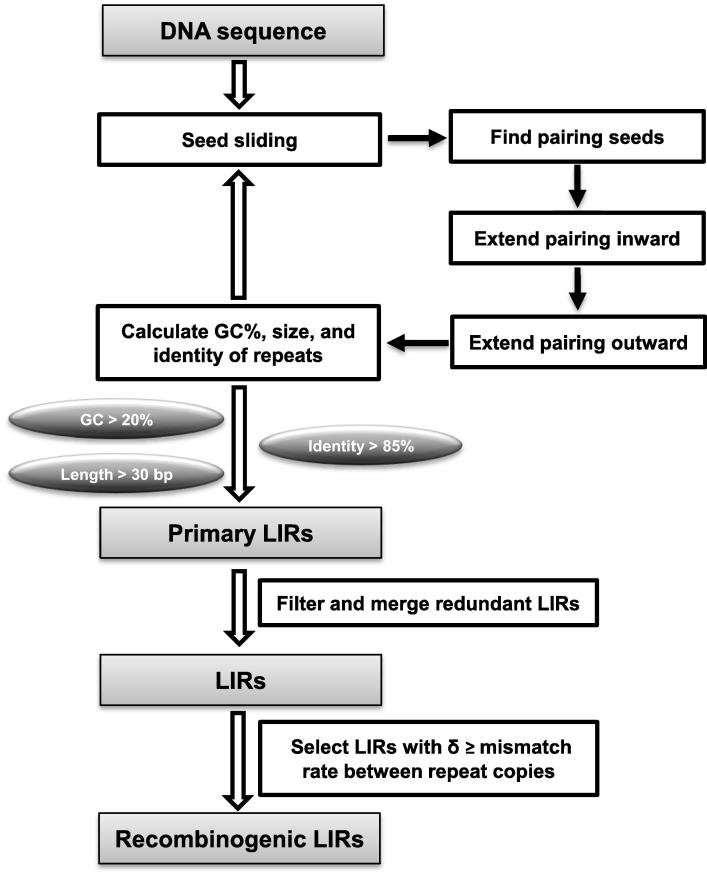
**Flowchart of LIR searching algorithm in Lirex** For a pair of seeds located in a DNA window of a defined size, pairing seeds are extended both inward and outward. If seeds extend to >30 bp with GC >20% and repeat identity >85%, the seeds are considered as the primary LIRs. A final list of LIRs was generated after filtering and merging the redundant LIRs. When the ratio of repeat length to internal spacer, *δ*, is greater or equal to mismatch rate between repeat copies, the LIR is considered to be recombinogenic. LIR, long inverted repeat.

Lirex is a Java tool and can be implemented on different systems, including Windows, Mac, and Linux. To identify an LIR, a pair of short fragments (also called “seed”) should be located in a DNA window of a defined size. Once pairing counterpart of a seed is found, both ends of the seed would be extended to search for pairing nucleotides with the counterpart (Figure 1). The inward extension terminates when there is no spacer between the seed and its pairing counterpart. One mismatch or insertion/deletion (indel) is permitted if subsequent pairing allows the extension to continue. There is no length limitation for outward extension until it is terminated by two continuous mismatches or indels. If the seed is extended to >30 bp, GC content and mismatch rate (N/L; N refers to the number of mismatches and L represents the length of stem) of the seed will be calculated. GC content is calculated to exclude the low complexity LIRs like (TA)n. If the mismatch rate is <15% (*i.e.*, identity of repeats >85%) and GC content is >20%, a primary LIR is identified (Figure 1). Then a new round of search starts by positioning a new pairing oligonucleotide for the seed of the previous primary LIR at the 3′ downstream of the previous internal spacer. When the previous internal spacer is <5 bp, the sliding window will move to the 3′ end of the previous LIR and start a new round of LIR search.

**Figure 2:**
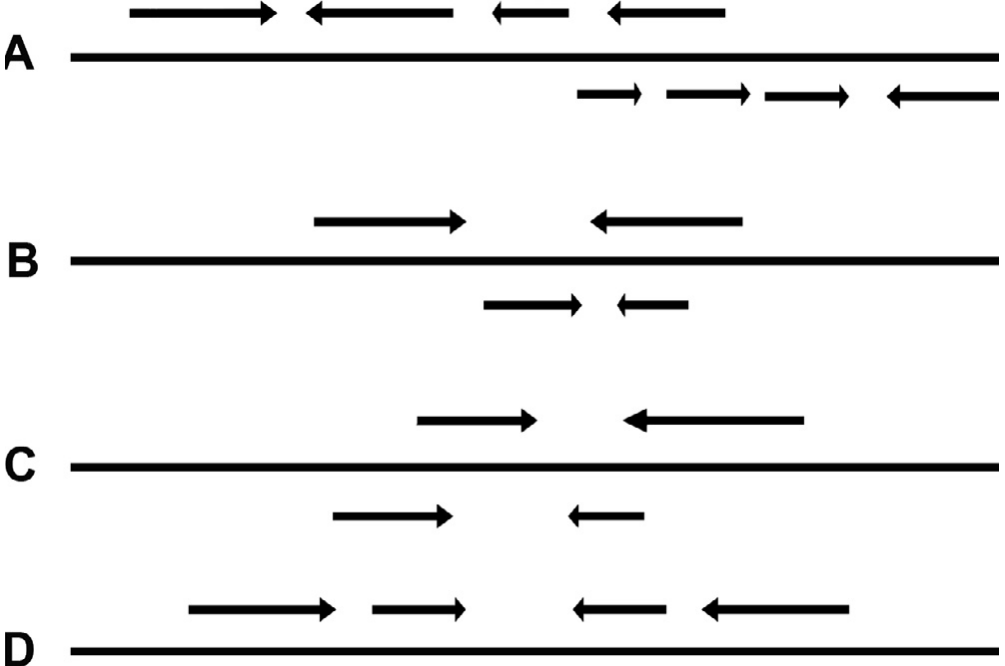
**Scenarios of redundancy in primary LIRs** Arrows denote copies of inverted repeats in genomic sequences. The four scenarios for the most frequent occurrences of redundant LIRs in the list of primary searching result are depicted in (A−D), respectively. LIR, long inverted repeat.

Second, redundancy in the list of primary LIRs will be removed. Occurrence of redundant LIRs is frequent in primate genomes, ascribed to the presence of repeats and transposons. There are four most common scenarios of redundancy (Figure 2): (1) overlap between the start or end side of the repeat copies of different LIRs; (2) a small repeat copy of one LIR is involved in the formation of a bigger repeat copy of another LIR; (3) overlap of two LIRs with repeat copies at the same sides shifted to some extent; and (4) one LIR lying completely in the spacer of another. To reduce the redundancies, additional filtering steps are designed to remove the primary LIRs falling into any of the four scenarios above. The basic rule is exclusion of the LIRs with longer internal spacers. For scenario (4), the two LIRs will be combined and counted as one LIR if their arms at the same side are separated by ≤5 bp.

Some LIRs may induce recombination in a genome. Lobachev et al. [4] have examined the inducing capacity of LIRs. They found that inverted *Alu* repeats separated <20 bp with identity >85% are labile. This indicates that internal spacer size and identity between repeat copies are the major factors attributable to recombination. In addition, the ratio of repeat length to internal spacer, termed as *δ*, is also critical. The recombinogenic ability is strong with *δ* > 16 [4]. Based on these findings, three major characteristics of LIRs (spacer size, repeat identity, and *δ*) are employed to select LIRs that may strongly induce recombination. When *δ* is greater or equal to mismatch rate between repeat copies, the LIR is predicted to be recombinogenic (*δ* is defined to be >1, when LIR copies are matched perfectly).

## Case study using a human genome sequence

**Figure 3:**
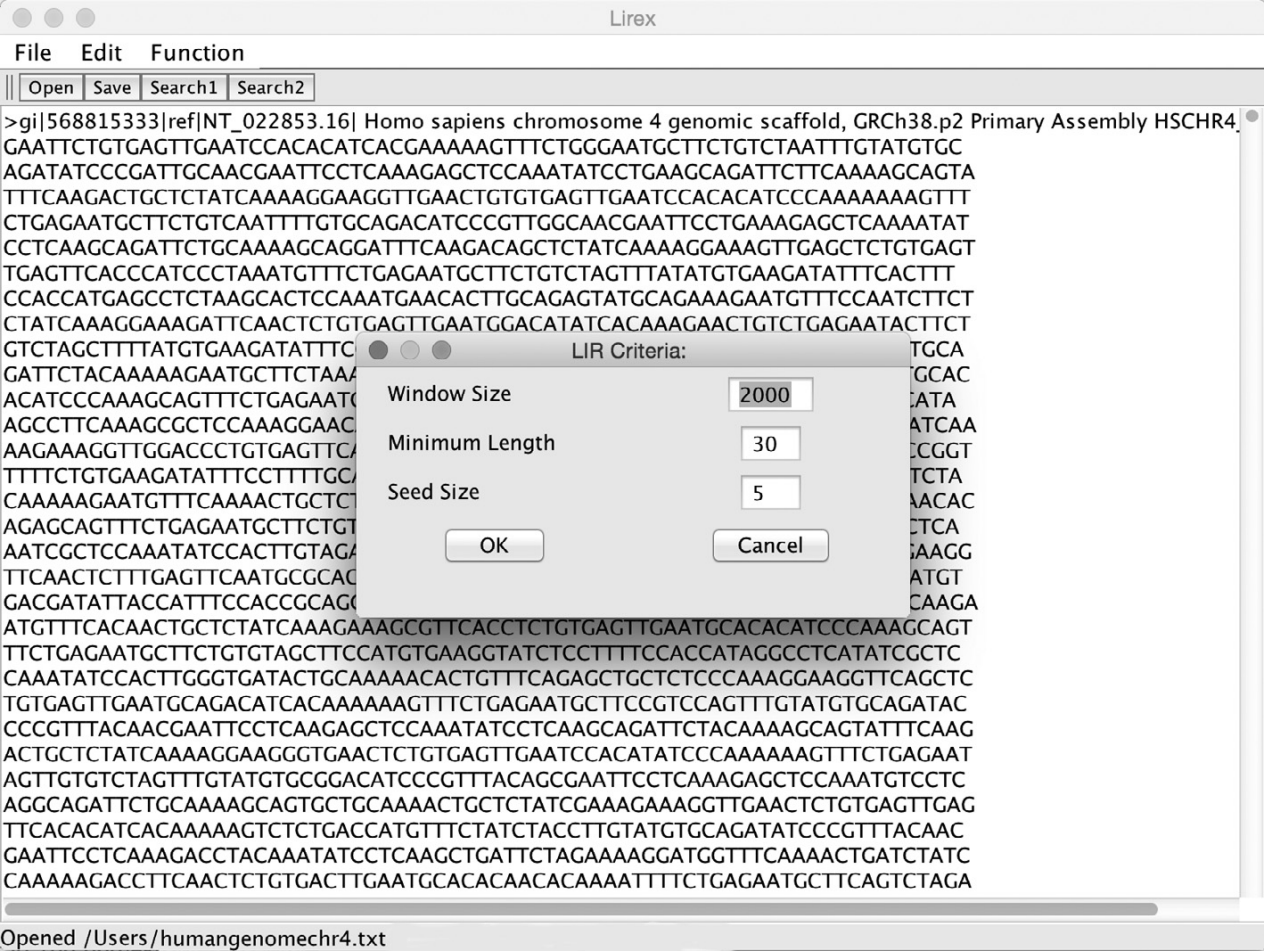
**Screenshot of the graphic user interface and criteria setting panel of Lirex** Lirex accepts DNA sequence in *Fasta* format. The setting panel is used for searching LIRs with unknown motifs in repeat copies. Window size refers to the maximum size of a sliding window in which primary LIRs are located. Minimum length with a default value of 30 bp is used to define the size of repeat copies in an LIR. Seed represents an oligonucleotide used to search for reversely complementary seed in a sliding window. LIR, long inverted repeat.

We applied the algorithm described above to develop Lirex, a Java multi-platform deployment tool for LIR exploration. Users are allowed to define criteria of LIRs such as sliding window size, repeat length, and seed size (Figure 3). If users have a known motif for one copy of the inverted repeats, a control panel may be used to specify the motif.

For demonstration, a contig in human chromosome 4 (NT_022853.16) was exemplified for LIR identification with default settings, *i.e.*, window size: 2000 bp; minimum length of repeat: 30 bp; and seed size: 5 bp. In total, 656 LIRs were identified in this contig and a partial list of the identified LIRs is shown in Table 1 (a complete list of LIRs identified is shown in Table S1). Some LIRs had a large internal spacer over 1 kb, although the corresponding repeat copies were shorter than 40 bp. Among 656 LIRs identified, four of them were considered to be recombinogenic (highlighted in bold in Tables 1 and S1).

In our previous studies, Lirex had been applied for a complete scan of the genomes of human and other model organisms [1], [14]. Over 100 recombinogenic LIRs were found in the human genome. Occasionally, one repeat of an LIR is located in an exon, exemplified by *GSTM5* gene [10]. Involvement of an exon in the formation of a stem-loop structure would result in even more complicated gene structures by creating various alternative splicing patterns, which has not been fully acknowledged at present. Therefore, more studies on LIRs in terms of their relative distance to other important genomic elements will deepen our understanding of the regulatory effect of LIRs as the source of structural complexity, which might be vital in mRNA structure and gene expression regulation.

## Performance comparison of LIR searching tools

We then compared the performance for predicting LIRs in NT_022853.16 (consisting of 7,084,842 bp) with different tools including Lirex, IRF, EMBOSS palindrome, detector, and MATLAB palindrome. The settings for prediction are the minimum LIR size of 30 bp, mismatch rate <15%, and the length of both stem and loop <2000 bp. A summary of the LIRs predicted by different tools is shown in Table 2. Lirex detected 1443 LIRs in the contig, including 28 perfect LIRs and 1415 imperfect LIRs. After filtration, there were still 656 LIRs. Obviously, Lirex outcompeted the other tools tested in this study by detecting much more LIRs. Moreover, Lirex could find the LIRs with mismatches or indels between the arms. Mismatch rate would be calculated later, rather than a setting of the maximal number of mismatches in detectIR [13]. On the other hand, Lirex took much longer running time, probably due to the detection of redundant LIRs in the scenarios shown in Figure 2.

## Conclusions

Lirex provides the users a powerful tool for easy identification of LIRs in a long genomic distance. The various secondary structures formed by the LIRs in transcripts may be then predicted based on the distribution of LIRs in a given gene. This will provide new insights into the genetic diseases and cancer induction. The algorithm will be optimized in future to shorten the time cost for the searching process.

## Authors’ contributions

YW designed the algorithm and the tool; YW and JMH wrote the manuscript and performed data analysis. Both authors read and approved the final manuscript.

## Competing interests

The authors have declared that no competing interests exist.

## Figures and Tables

**Table 1:** **A partial list of LIRs identified in a human contig (NT_022853.16) by Lirex**

**Position of left copy**	**Position of right copy**	**Repeat length**	**Mismatch rate**	**Size of internal spacer**
3801595…3801626	3801662...3801693	32	0.031	35
3808004...3808036	3809074...3809108	33	0.121	1037
3809309...3809356	3809684...3809731	48	0.083	327
3809775...3809812	3810283...3810317	35	0.142	470
3809920...3809972	3810588...3810640	53	0.056	615
3812968...3813016	3813948...3813994	47	0.148	931
3834774...3834814	3835410...3835450	41	0.024	595
3861385...3861419	3863345...3863379	35	0.085	1925
3868611...3868644	3869190...3869223	34	0.058	545
**3880525...3880559**	**3880580...3880614**	**35**	**0**	**20**
3892447...3892480	3894367...3894400	34	0.088	1886
3894385...3894435	3894952...3895002	51	0.058	516
**3903431...3903467**	**3903482...3903518**	**37**	**0**	**14**
3906855...3906912	3908736...3908793	58	0.068	1823
3970674...3970704	3971538...3971568	31	0.096	833
3979626...3979672	3979822...3979866	45	0.133	149
3989493...3989614	3990425...3990548	122	0.047	811

*Note*: The region between 3.8 Mb and 4.0 Mb of NT_022853.16 on human chromosome 4 was selected for demonstration with the positions of left and right copies of the LIRs indicated. Mismatch rate was calculated as the ratio of the number of mismatches between the two LIR repeat copies to the length of one of the repeat copies. The recombinogenic LIRs are indicated in bold. LIR, long inverted repeat.

**Table 2:** **Performance comparison of LIR identification in NT_022853 with different tools**

**Parameter**	**Lirex**	**IRF**	**EMBOSS palindrome**	**detectIR**	**MATLAB palindrome**
Setting	Window size: 2000 bp Minimum length: 30 bp Seed size: 5 bp	T4 class Maximum search distance: 74 bp T5 class Maximum search distance: 493 bp T7 class Maximum search distance: 10,000 bp Maximum loop: 2000 bp Maximum stem: 10,000 bp	For perfect LIRs minpallen: 30 bp maxpallen: 1000 bp gaplimit: 0 For imperfect LIRs minpallen: 30 bp maxpallen: 1000 bp gaplimit: 2 bp	For perfect LIRs minLen: 60 bp maxLen: 2000 bp For imperfect LIRs minLen: 60 bp maxLen: 2000 bp maxMismcNum: 6 bp	For perfect LIRs Length: 60 bp For imperfect LIRs Length: 60 bp Gap: 2 bp

Running time	∼9 h	1 min	14.2 min + 39.2 min	1.5 s + 13 s	2.9 s + 7.2 s

Number of perfect LIRs	28	7	8	19	7

Number of imperfect LIRs	1415	198	419	324	8

Total	1443	205	427	343	15

*Note*: The complete sequence of NT_022853.16 was examined for identification of LIRs using different tools. For detectIR and MATLAB palindrome, length refers to the sum of both stem size and loop size. LIR, long inverted repeat.

## Supplementary material

Supplementary Table S1 **A complete list of LIRs identified in a human contig (NT_022853.16) by Lirex**
